# Predictive factors for treatment failure in reducing alcoholic consumption by case management in alcoholics living in permanent rental apartments

**DOI:** 10.1192/j.eurpsy.2022.2158

**Published:** 2022-09-01

**Authors:** S.-G. Kim, S. Huh, Y.S. Kim

**Affiliations:** 1 Pusan National University Yangsan Hospital, Psychiatry, Yangsan, Korea, Republic of; 2 Pusan National University School of Medicine, Psychiatry, Yang-San , Korea, Republic of; 3 Pusan National University Yangsan Hospital, Psychiatry, YangSan, Korea, Republic of

**Keywords:** alcoholism, case management, predictive factor, treatment failure

## Abstract

**Introduction:**

Based on the results showing that there are more alcoholics in the low-income bracket, case management (CM) for such cases was initiated in 2011. As a result, the treatment failure rate was identified between 43-44% based on the WHO criteria.

**Objectives:**

We investigated the predictive factors for the treatment failure to maximize successful CM treatment.

**Methods:**

Thirty-nine subjects from Sasang-gu and Saha-gu treated by four social workers using CM were included in this study. Failure was defined when the level of risk was maintained or increased as per the WHO criteria. The clinical characteristics of the subjects including their age and gender were collected.

**Results:**

Typically, 17 (43.6%) subjects demonstrated treatment failure by the CM (TF). Compared with the subjects who were treated successfully (n=22; TS), TF maintained abstinence in shorter periods in terms of the longest abstinent period compared with TS while CM (28.24±.99 vs. 76.82±.27, p=.025). The higher population in TF did not make an effort to quit drinking compared with TS while CM (41.2% vs.13.6%, p=.051). Also, more TF stayed with their family members compared with TS (58.8% vs. 31.8%, p=.092).

**Conclusions:**

The results showed that shorter abstinence periods and the absence of efforts initiated to quit drinking while CM, and living with family members were the predictive factors for failure in treating alcoholics by the CM. It is presumed that influencing patients to quit drinking and encouraging them to abstain for longer periods are crucial to attaining successful treatment.

**Disclosure:**

No significant relationships.

